# Anti-biofilm Activity of Oral Health-care Products Containing Chlorhexidine Digluconate and Citrox

**DOI:** 10.3290/j.ohpd.a45437

**Published:** 2020-10-27

**Authors:** Jenaniy Jeyakumar, Anton Sculean, Sigrun Eick

**Affiliations:** a Dentist, Department of Periodontology, School of Dental Medicine, University of Bern, Bern, Switzerland. Performed the experiments in partial fulﬁllment of requirements for a DMD, wrote and proofread the manuscript.; b Professor, Department of Periodontology, School of Dental Medicine, University of Bern, Bern, Switzerland. Idea, contributed substantially to discussion, proofread the manuscript.; c Professor, Department of Periodontology, School of Dental Medicine, University of Bern, Bern, Switzerland. Idea, experimental design, analysis, wrote and proofread the manuscript.

**Keywords:** biofilm, caries, chlorhexidine digluconate, mouthrinse, periodontitis

## Abstract

**Purpose:**

To analyze in vitro new formulations with Citrox and chlorhexidine digluconate (CHX) regarding their antibacterial activity against planktonic bacteria and their potential to inhibit biofilm formation or to act on existing biofilms.

**Materials and Methods:**

Five oral health care products with 0.05%–0.5% CHX formulations (four rinses and one gel) were compared with Citrox preparations and additive-free CHX solutions. The minimal inhibitory concentrations (MIC) were determined against 13 oral bacteria associated with caries or periodontitis. Further, the activity on retarding biofilm formation and on existing biofilms was analyzed; both a ‘cariogenic’ (5 species) and a ‘periodontal’ (12 species) biofilm were included.

**Results:**

The MIC values did not differ between the CHX mouthrinse/gel formulations and the respective additive-free CHX solutions. Citrox was active against selected periodontopathogens (e.g. *Porphyromonas gingivalis*). The CHX formulations more effectively retarded biofilm formation than did solutions with the same concentration of CHX but without additives. The anti-biofilm activities depended on the CHX concentration in the formulations. Both CHX solutions and formulations (rinse and gel) were only slightly active on an already formed biofilm. Citrox did not exert any anti-biofilm effect.

**Conclusion:**

The present in vitro data support the anti-biofilm activity of the novel CHX, Citrox, poly-L-lysine and xylitol oral health-care formulations. Further studies are warranted to confirm the present findings in various clinical settings.

Oral health-care products are widely used in prevention and treatment of oral diseases caused by biofilms. Among the antiseptics, products containing chlorhexidine digluconate (CHX) are still the gold standard.^29^ As recently reviewed, the beneficial effects of CHX are confirmed for reducing plaque accumulation, and in dental caries, gingivitis, periodontitis.^17^ Adjunctive use of CHX mouthrinses in non-surgical periodontal therapy results in additional probing depth reduction.^4^ Using 0.12% CHX solution is recommended for high-caries-risk patients.^7^ During fixed orthodontic therapy, CHX varnishes are effective in reducing caries incidence.^25^

However, the CHX formulations have different side-effects, e.g. external tooth staining, taste alterations, and burning sensations.^15^ To limit side-effects, CHX formulations may include certain additives. In part, these additives interfere with the action of CHX. Some in vitro studies have shown that CHX mouthrinses containing an anti-discoloration system (ADS) were less active than other CHX preparations against planktonic bacteria^5^ and growing biofilm.^11^ In an in vivo study,^10^ three 0.2% CHX formulations were compared: one with ADS, one with ethanol, and one without ADS and ethanol; the formulation with ADS less effectively reduced plaque, and the formulation with ethanol was less effective in reducing gingival inflammation.

In several in vitro studies, the cytotoxicity of CHX has been demonstrated.^21^,^24^ The toxicity clearly depends on the concentration. Human fibroblasts and osteoblasts tolerate concentrations less than 0.02%,^21^ whereas 0.2% CHX showed a strong and 0.05% CHX a moderate cytotoxicity against gingival fibroblasts.^24^ Thus, due to the reported adverse effects and the potential cytotoxicity, there is a need to develop formulations lacking or containing a reduced concentration of CHX that might be as effective as solutions containing 0.12% or 0.2% CHX. Citrox was proposed as a potential alternative or supplement. It is derived from citrus fruits, contains many different bioflavonoids, and is used as an additive to commercial sanitizers^22^ and food products.^32^

In the present study, new formulations with Citrox and CHX at concentrations from 0.05% to 0.2% were evaluated in vitro regarding their antibacterial activity against planktonic bacteria and their potential to inhibit biofilm formation or act on existing biofilms. The biofilms included bacteria associated either with caries or periodontal disease. The study question was whether these formulations are equally or even more active as a solution with the same % of CHX and without additives.

## Materials and Methods

### CHX Formulations

The experiment included five oral health care products with CHX, four rinsing formulations and one gel (all obtained from CURADEN; Kriens, Switzerland). The mouthrinses contained 0.2% CHX (CHX0.2C, Curaprox PerioPlus forte), 0.12% CHX (CHX0.12C; Curaprox PerioPlus Protect), 0.09% CHX (CHX0.09C, Curaprox PerioPlus Regenerate) and 0.05% CHX (CHX0.05C; Curaprox PerioPlus Balance). A gel formulation with 0.5 CHX (CHX0.5Cg) completed the tested oral health care products. Besides CHX, Citrox and poly-L-lysine were also constituents of all the formulations. Further, all the oral health care products contained xylitol and PVP-VA. Hyaluronic acid and cyclodextrin were added to the CHX0.09C formulation, the CHX0.05C formulation was supplemented with sodium fluoride and the CHX0.5Citgel with hyaluronic acid.

As controls, two Citrox preparations one without (Cit) and one with poly-L-lysine (CitPLL) were used. The negative control was 0.9% w/v NaCl solution; the positive controls were CHX solutions without additives at three CHX concentrations (0.2% [CHX0.2]; 0.12% [CHX0.12] and 0.05 % [CHX0.05]).

### Microorganisms

Fifteen different bacterial strains were used in the experiments:

- *Streptococcus gordonii* ATCC 10558
- *Actinomyces naeslundii* ATCC 12104
- *S. mutans* ATCC 25175
- *S. sobrinus* ATCC 33478
- *Lactobacillus acidophilus* ATCC 11975
- *Fusobacterium nucleatum* ATCC 25586
- *Campylobacter rectus* ATCC 33238
- *Parvimonas micra* ATCC 33270
- *Eikenella corrodens* ATCC 23834
- *Prevotella intermedia* ATCC 25611
- *Capnocytophaga gingivalis* ATCC 33624
- *Porphyromonas gingivalis* ATCC 33277
- *Tannerella forsythia* ATCC 43037
- *Filifactor alocis* ATCC 33099
- *Treponema denticola* ATCC 35405.

Except for *F. alocis* and *T. denticola*, minimal inhibitory concentration (MIC) values of the formulations and controls were determined against all other strains. ‘Cariogenic’ biofilm was formed of all streptococcal strains, *A. naeslundii* ATCC 12104 and *L. acidophilus* ATCC 11975. The ‘periodontal’ biofilm consisted of *S. gordonii* ATCC 10558, *A. naeslundii* ATCC12104, *Fusobacterium nucleatum* ATCC 25586, *C. rectus* ATCC 33238, *P. micra* ATCC 33270, *E. corrodens* ATCC 23834, *P. intermedia* ATCC 25611, *C. gingivalis* ATCC 33624, *P. gingivalis* ATCC 33277, *T. forsythia* ATCC 43037, *F. alocis* ATCC 33099, and *T. denticola* ATCC 35405. The strains were passaged on tryptic-soy agar plates (Oxoid; Basingstoke, GB) with 5% sheep blood and with 10 mg/l N-acetylic muramic acid for *T. forsythia*. *T. denticola* ATCC 35405 was maintained in modified mycoplasma broth (BD; Franklin Lakes, NJ, USA) enriched with 1 mg/ml glucose, 400 μg/ml niacinamide, 150 μg/ml spermine tetrahydrochloride, 20 μg/ml Na isobutyrate, 1 g/ml cysteine, and 5 μg/ml cocarboxylase. All chemicals were bought from Merck (Darmstadt, Germany). All the strains were cultured at 37°C, with streptococci, *A. naeslundii* ATCC 12104 and *L. acidophilus* ATCC 11975 cultured in 10% CO_2_, and the other strains under anaerobic conditions.

### Determination of MIC

The microbroth dilution technique was used to determine MIC values. After subcultivation of bacterial strains and purity checking, a defined inoculum was added to Wilkins-Chalgren broth (Oxoid) supplemented with 10 µg/ml β-NAD and defined concentrations of the formulations (starting from 10% of the final formulations). After an incubation time of 42 h (18 h for aerobic strains), the growth of microbes was analyzed by visual checking of turbidity (and if necessary, by subcultivation). MIC represented the lowest concentration without visible turbidity.

These experiments were performed in independent replicates.

### Activity on Biofilms

Two different experimental designs were conducted: (a) the application of mouthrinse after mechanical removal of biofilm to show the influence on biofilm formation, and (b) application on an established biofilm.

#### Activity on biofilm formation

The formulations and solutions were diluted to a 10% concentration with dH_2_O. The wells of four 96-well plates were coated with 25 µl of test substances. After 30 min of incubation, 25 µl/well protein solution (1.5% bovine serum albumin in PBS) were added for another 30 min. Bacteria were suspended in 0.9% w/v NaCl according to the McFarland standard 0.5. Then the suspensions for the respective biofilms were mixed together, in each case one part *S. gordonii* ATCC10558, two parts *A. naeslundii* ATCC 12104 and four parts each of the other bacterial strains. Subsequently (time 0 h), 200 µl of bacterial suspension mixed with nutrient broth (Wilkins-Chalgren broth supplemented with 10 µg/ml β-NAD and 10 mg/l N-acetylic muramic acid for the ‘periodontal’ biofilm) in a volume ratio of 1:9 were added. After 6 h and 24 h of incubation in the respective atmosphere (cariogenic biofilm with 10% CO_2_, ‘periodontal’ biofilm under anaerobic conditions), the nutrient broth was carefully removed and the biofilms were washed briefly with 0.9% w/v NaCl. Then biofilms (one 96-well-plate each at the designated time) were scraped from the surface and suspended in 0.9% w/v NaCl and, after making a dilution series, plated on tryptic-soy agar plates. After incubation under the respective conditions, the colony forming units (CFU) were counted. At 24 h from the third 96-well-plate, the biofilms were quantified after staining with crystal violet according to recently published protocols.^18^ From the fourth plate, the metabolic activity of the biofilm suspension was assessed using Alamar blue as a redox indicator.^26^

#### Established biofilm

In each experiment, three 96-well plates were used. The wells of the 96-well plates were coated with 25 µl/well protein solution (1.5% bovine serum albumin in PBS) for 30 min. Then, the bacteria/nutrient broth mixture was prepared as described above and 225 µl were pipetted into each well. The plates were incubated in the respective atmosphere for 48 h. Subsequently, in the case of the periodontal biofilm, 10 µl each of *P. gingivalis* ATCC 33277, *T. forsythia* ATCC 43037 and *T. denticola* ATCC 35405 were added per well, and these plates were incubated for another 36 h. At 48 h for the ‘cariogenic’ biofilm and at 3.5 days for the ‘periodontal’ biofilm, the meanwhile established biofilms were treated with 25 µl of the test substances for 1 min after removing nutrient broth and washing briefly. After 1 min, nutrient broth (225 µl) was added and the biofilms were incubated for 1 h. Analysis was then performed as described above, i.e. CFU count, biofilm mass and metabolic activity.

### Statistical Analysis

**Fig 1:**
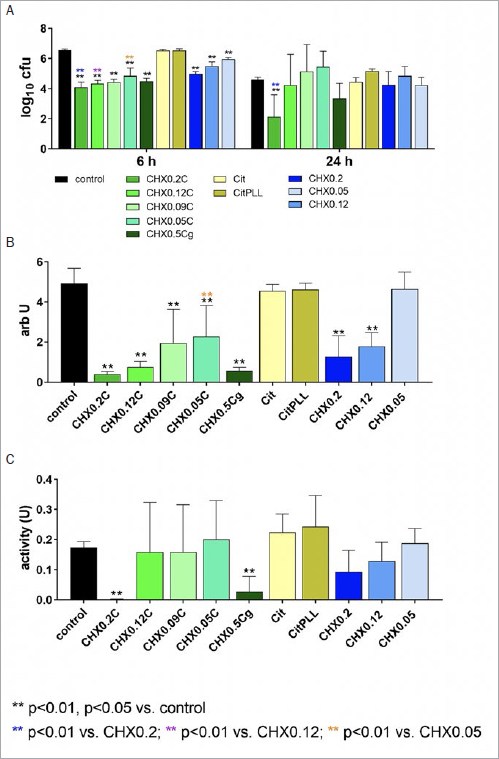
Activity of different formulations/controls (coating the surface with 10%, final concentration in the assays 1% of the formulations/solutions) on bacterial counts (A) after 6 h and 24 h of incubation, mass (B), and metabolic activity (C), both at 24 h in the formation of cariogenic biofilm consisting of five different species. Tested formulations with CHX, Citrox and poly-L-lysine: mouthrinses with 0.2% CHX (CHX0.2C), 0.12% CHX (CHX0.12C), 0.09% CHX (CHX0.09C) and 0.05% CHX (CHX0.05C) and a gel formulation with 0.5 CHX (CHX0.5Cg). Controls: 0.9% w/v NaCl as negative control (control); Citrox preparations without (Cit) and with poly-L- lysine (CitPLL), additive-free CHX solutions as positive controls with 0.2% CHX (CHX0.2), 0.12% CHX (CHX0.12) and 0.05 % CHX (CHX0.05).

**Fig 2:**
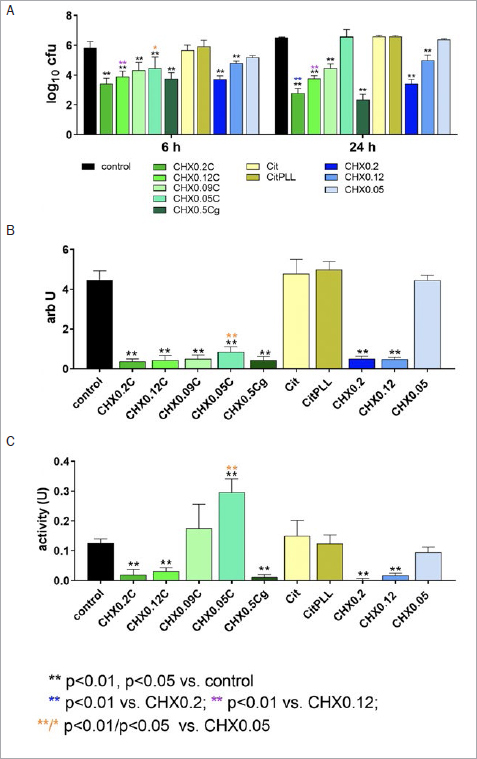
Activity of different formulations/controls (coating of the surface with 10%, final concentration in the assays 1% of the formulations/solutions) on bacterial counts (A) after 6 h and 24 h of incubation, mass (B), and metabolic activity (C) both at 24 h in the formation of periodontal biofilm consisting of 12 different species. Tested formulations with CHX, Citrox and poly-L-lysine: mouthrinses with 0.2% CHX (CHX0.2C), 0.12% CHX (CHX0.12C), 0.09% CHX (CHX0.09C) and 0.05% CHX (CHX0.05C) and a gel formulation with 0.5 CHX (CHX0.5Cg). Controls: 0.9% w/v NaCl as negative control (control); Citrox preparations without (Cit) and with poly-L- lysine (CitPLL), additive-free CHX solutions as positive controls with 0.2% CHX (CHX0.2), 0.12% CHX (CHX0.12) and 0.05 % CHX (CHX0.05).

**Fig 3:**
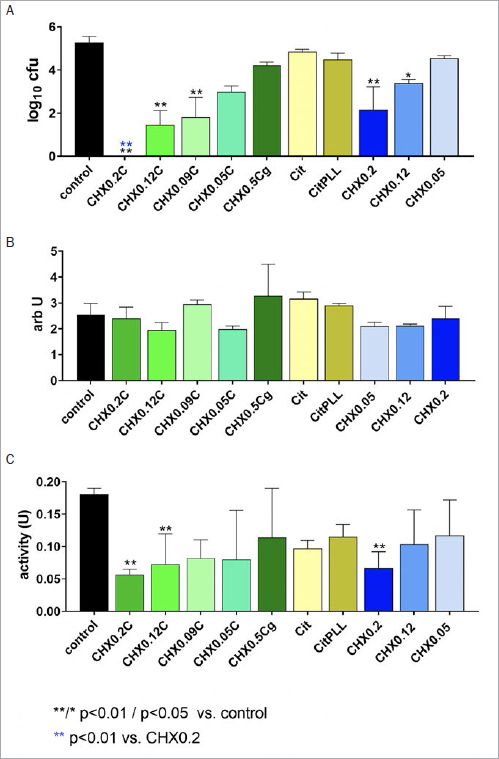
Activity of different formulations/controls on bacterial counts (A), mass (B), and metabolic activity (C) of the established cariogenic biofilm formed by five bacterial species for 48 h and after 1 h of exposition (1 min with 100% of the formulation/solution, thereafter 10% for 1 h). Tested formulations with CHX, Citrox and poly-L-lysine: mouthrinses with 0.2% CHX (CHX0.2C), 0.12% CHX (CHX0.12C), 0.09% CHX (CHX0.09C) and 0.05% CHX (CHX0.05C) and a gel formulation with 0.5 CHX (CHX0.5Cg). Controls: 0.9% w/v NaCl as negative control (control); Citrox preparations without (Cit) and with poly-L-lysine (CitPLL); additive-free CHX solutions as positive controls with 0.2% CHX (CHX0.2), 0.12% CHX (CHX0.12) and 0.05 % CHX (CHX0.05).

**Fig 4:**
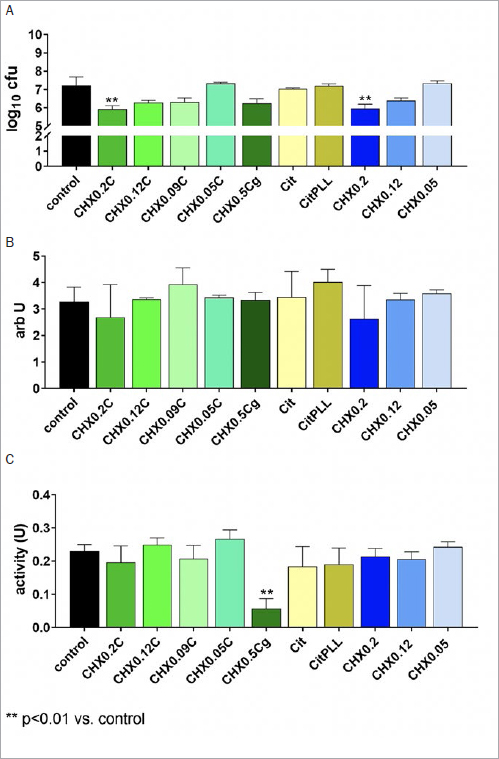
Activity of different formulations/controls on bacterial counts (A), mass (B), and metabolic activity (C) of the established periodontal biofilm formed by 12 bacterial species for 3.5 days and after 1 h of exposition (1 min with 100% of the formulation/solution, thereafter 10% for 1 h). Tested formulations with CHX, Citrox and poly-L-lysine: mouthrinse formulations with 0.2% CHX (CHX0.2C), 0.12% CHX (CHX0.12C), 0.09% CHX (CHX0.09C) and 0.05% CHX (CHX0.05C), and a gel formulation with 0.5 CHX (CHX0.5Cg). Controls: 0.9% w/v NaCl as negative control (control); Citrox preparations without (Cit) and with poly-L-lysine (CitPLL); CHX solutions without additives as positive controls with 0.2% CHX (CHX0.2), 0.12% CHX (CHX0.12) and 0.05 % CHX (CHX0.05).

Statistical analysis was conducted using SPSS 26.0 (IBM; Chicago, IL, USA). These biofilm experiments were carried out as two independent series in each independent quadruplicate (8 single values). CFU counts were recorded as log10 CFU. For statistical analysis, ANOVA was performed first. Given statistical significance, the post-hoc Bonferroni test was done to record results. In Figs 1 to 4 (below), each statistically significant difference vs the controls as well as between the CHX formulation and its respective solution (CHX0.2C vs CHX0.2, CHX0.12C vs CHX0.12 and CHX0.05C vs CHX0.05) are given. A p-value of 0.05 was considered statistically significant.

## Results

### MIC Values

Comparing the MIC values of the CHX mouthrinses with the respective CHX solutions, the difference did not exceed one dilution step. The only exception was *L. acidophilus,* which was more susceptible to the CHX solutions than to the CHX formulations. The Citrox preparations were most active against *P. gingivalis* ATCC 33277, and moderately antibacterial against *F. nucleatum* ATCC 25586, *P. micra* ATCC 33270, and *C. gingivalis* ATCC 33624. Against all other strains, the MICs were 5% or higher of the Citrox formulations. Adding poly-L-lysine made no statistically significant difference (Table 1).

**Table 1:** Minimum inhibitory concentrations of oral health care products (formulations) and CHX solutions (MIC% of the respective formulation/solution; tested in the 0.16% – 10% range)

Strain	CHX0.2C	CHX0.12C	CHX0.09C	CHX0.05C	CHX0.5Cg	Cit	CitPLL	CHX0.2	CHX0.12	CHX0.05
*Streptococcus gordonii* ATCC 10558	≤0.16	≤0.16	≤0.16	≤0.16	≤0.16	>10	>10	≤0.16	≤0.16	≤0.16
*Actinomyces naeslundii* ATCC 12104	≤0.16	≤0.16	≤0.16	0.31	≤0.16	>10	>10	≤0.16	0.31	0.63
*S. mutans* ATCC 25175	≤0.16	≤0.16	≤0.16	≤0.16	≤0.16	>10	>10	≤0.16	≤0.16	≤0.16
*S. sobrinus* ATCC 33478	≤0.16	≤0.16	≤0.16	≤0.16	≤0.16	>10	>10	≤0.16	≤0.16	≤0.16
*Lactobacillus acidophilus* ATCC 11975	1.25	1.25	1.25	10	≤0.16	>10	>10	≤0.16	≤0.16	≤0.16
*Fusobacterium nucleatum* ATCC 25586	≤0.16	≤0.16	≤0.16	0.31	≤0.16	1.25	1.25	≤0.16	≤0.16	0.31
*Campylobacter rectus* ATCC 33238	≤0.16	≤0.16	≤0.16	≤0.16	≤0.16	5	5	≤0.16	≤0.16	≤0.16
*Parvimonas micra* ATCC 33270	0.63	0.31	0.63	0.63	≤0.16	1.25	1.25	0.31	0.31	0.63
*Eikenella corrodens* ATCC 23834	≤0.16	≤0.16	≤0.16	0.31	≤0.16	10	10	≤0.16	≤0.16	≤0.16
*Prevotella intermedia* ATCC 25611	≤0.16	≤0.16	≤0.16	≤0.16	≤0.16	5	10	≤0.16	≤0.16	0.31
*Capnocytophaga gingivalis* ATCC 33624	0.31	0.63	0.63	1.25	≤0.16	1.25	1.25	≤0.16	0.63	1.25
*Porphyromonas gingivalis* ATCC 33277	≤0.16	≤0.16	≤0.16	0.31	≤0.16	0.63	0.63	≤0.16	≤0.16	≤0.16
*Tannerella forsythia* ATCC 43037	≤0.16	≤0.16	≤0.16	0.31	≤0.16	5	5	≤0.16	0.31	0.63

### Activity of CHX Formulations on Biofilm Formation

According to the protocol, the final concentration in the assay was 1% of the formulation.

In the case of the ‘cariogenic’ biofilm, all CHX containing formulations and solutions statistically significantly reduced the CFU counts vs control at 6 h (p<0.001). The highest reductions were seen for CHX0.2C both after 6 h (-2.45 log10 CFU) and 24 h (-2.24 log10 CFU) of biofilm formation. At 6 h, the CFU counts were lower for the mouthrinse formulations. (CHX0.2C, CHX0.12C and CHX0.05C) compared with their respective CHX controls (CHX0.2, CHX0.12 and CHX0.05; p<0.001). It is of interest to note that the low-concentration formulations reduced CFU counts to greater extents than the higher-concentration CHX solutions, e.g. CHX0.09C was more active than CHX0.12 (-1.13 log10, p<0.001) and even moreso than CHX0.2 (-0.56 log10, p=0.001). At 24 h, only the counts after applying CHX0.2C were less than those of the control (p<0.001). The difference vs CHX0.2 was also statistically significant (p<0.001). The Citrox formulations did not affect the CFU counts at any time (Fig 1A).

The biofilm mass of the cariogenic biofilm after 24 h of formation clearly depended on the CHX concentration in the formulations and solutions. The differences were statistically significant for all CHX formulations and the CHX0.2 and CHX0.12 solutions vs the controls (p<0.001). The biofilm mass was lower after CHX0.05C than after CHX0.05 (p<0.001) (Fig 1B).

The metabolic activity was only reduced after applying CHX0.2C and CHX0.5Cg (p<0.001 vs control) (Fig 1C).

In the case of the ‘periodontal’ biofilm all formulations and solutions containing ≥ 0.09% CHX statistically significantly reduced the CFU counts vs control (p<0.001) at 6 h and 24 h of biofilm formation. After 6 h, there was also a statistically significant difference between CHX0.05C and the control (p<0.001). The greatest reductions were seen for CHX0.2C after 6 h (-2.42 log10 CFU) and for CHX0.5Cg after 24 h (-4.16 log10 CFU) of biofilm formation. At 6 h, the CFU counts were lower for the mouthrinse formulations CHX0.12C and CHX0.05C in comparison with their respective control solutions CHX0.12 (p=0.001) and CHX0.05 (p=0.019). At 24 h, the counts after applying CHX0.2C and CHX 0.12C were lower than those of the solutions CHX0.2 and CHX0.12 (p<0.001), and those after CHX0.09C were also reduced to a greater extent than after CHX0.12 (p<0.001). The Citrox formulations did not affect the CFU counts (Fig 2A).

The biofilm mass of the ‘periodontal’ biofilm after 24 h was lower after applying any of the CHX formulations or CHX0.2 and CHX0.12 (p<0.001). CHX0.05C produced a greater reduction in biofilm mass than did CHX0.05 (p<0.001) (Fig 2B).

The metabolic activity decreased after applying CHX0.2C, CHX0.12C, CHX0.5Cg and CHX0.2 and CHX0.12 (p<0.001 vs control). It increased after applying CHX0.05C (p<0.001) (Fig 2C).

### Activity of CHX Formulations on Established Biofilm

Differences between the two biofilm models were apparent. The cariogenic biofilm controls contained a mean of 5.26 log10 CFU, those of the periodontal biofilm 7.22 log10.

In the cariogenic biofilm, CHX mouthrinse formulations and solutions with ≥0.09% CHX statistically significantly reduced the CFU counts (CHX0.2C, CHX0.12C: p<0.001; CHX0.09C: p=0.008; CHX0.2: p=0.001; CHX0.12: p=0.019). CHX0.2C was the most active, as no CFU were counted after application. The difference to CHX0.2 was statistically significant (p=0.001). The Citrox formulations without CHX did not affect the CFU counts (Fig 3A). An influence on biofilm mass was not found for any of the formulations or controls (Fig 3B). The metabolic activity decreased after application of CHX0.2C (p=0.009), CHX0.12C (p=0.002) and CHX0.2 (p<0.001) (Fig 3C).

In the periodontal biofilm, only the CHX mouthrinse formulation and solution with 0.2% CHX statistically significantly decreased the CFU counts. The difference of CFU counts were -1.31 log10 (p=0.009) for CHX0.2C and -1.26 log10 (p=0.001) for CHX0.2 (Fig 4A). An influence on biofilm mass was not found (Fig 4B), and the metabolic activity decreased only after application of CHX0.5Cg (p<0.001) (Fig 4C).

## Discussion

The present results demonstrated that the new CHX formulations tested here were active against the selected oral bacteria. They retarded biofilm formation to a greater extent than additive-free solutions with the same concentration of CHX. The anti-biofilm activities depended on the CHX concentration of the formulations. However, as also for the tested solutions, the formulations only had minor activity on already extant biofilm.

In the present study, two different biofilm models and two different approaches were used. The biofilm models were designed to resemble caries and a periodontal disease. Defined strains were used to allow reproducible experiments with standardized conditions. One limitation of our study is the biofilm model used. The use of multispecies biofilms implies interaction between the various constituative species, but it does not reflect the complexity present in the oral cavity, which consists of substantially more microorganism species. Using modern technologies, about 70 different microorganisms in caries^2^ and about 300 in periodontal disease^20^ have been identified. Further limitations are the application and use of a static model. In the case of biofilm formation, the formulations/solutions were applied only once and there was a constant concentration of 1% of the respective formulation/solution in the assay. In the established model, a 100% concentration of the formulations and solutions were applied for a short time before diluting to 10%. Limitations of the static biofilm are also visible in the cariogenic biofilm model. When the different biofilms were formed, the log10 CFU counts of the cariogenic biofilm were higher after 6 h than after 24 h, whereas in case of the periodontal biofilm, there was a continued increase. The cariogenic biofilm produced here chiefly consisted of streptococci, whereas anaerobic bacteria were dominant in the periodontal biofilm. The doubling time of streptococci is much shorter (4 – 6 h) compared with that of gram-negative anaerobes (20- 24 h),^23^ suggesting that bacteria in the cariogenic biofilm model more rapidly consumed the available nutrients. Thus, the results obtained after 6 h of cariogenic biofilm formation might more closely resemble an in vivo situation.

Citrox was one of the additives in the tested formulations. It derives from citrus fruits, contains many different bioflavonoids, and was first used as an additive in a commercial sanitizer.^22^ Citrox is also present as a food additive, where it decreases the counts of certain pathogens such as *Salmonella* sp.^32^ It is also active against *Staphylococcus aureus* strains and reduces the viability of biofilms.^13^ Good to moderate activity was also found against oral microorganisms.^14^ However, the results of the present study were different. MIC values were higher against oral streptococci and *Actinomyces* ssp., but lower against *P. gingivalis,* which may depend on the cultivation media used. Further, no activity of Citrox against biofilm formation or an established biofilm was observed in our experiments. One explanation for this finding might be that in the present study, more complex multispecies biofilm models were used.

Although no effect was found for Citrox, the formulations were shown to inhibit biofilm formation. Even the low-concentration CHX formulations slowed cariogenic biofilm formation to a greater extent than did higher-concentration CHX solutions without additives. This effect might be related to constituents other than Citrox. All the formulations contained xylitol and poly-L-lysine. Xylitol has been described to be an anti-adherent agent in biofilm formation.^8^ In vitro, it inhibited formation of single-species biofilms of *S. mutans* and *S. sobrinus*^28^ and also that of a dual-species biofilm of *S. gordonii* and *P. gingivalis*.^12^ Poly-L-lysine has a strong antibacterial and anti-biofilm activity against *S. aureus*.^1^ Functionalized titanium surfaces with poly-L-lysine containing silver nanoparticles showed enhanced antimicrobial activity.^9^ The effect was explained by the binding of poly-L-lysine to the negatively charged nanoparticles.^9^ This cannot be assumed for binding to CHX, as this is positively charged.^17^ However, there might be a synergistic effect of binding to negatively charged surfaces such as teeth and probably the plastic surfaces of microtiter plates.

As recently stated in a systematic review, despite the fact that CHX mouthrinses reduce *S. mutans* counts in saliva, a definitive conclusion on its efficacy in preventing new caries lesions could not be drawn.^3^ The efficacy of CHX mouthrinses on the reduction of *S. mutans* depends on their concentration,^16^ which was confirmed by the present in vitro study. Fluoride supplementation to CHX solution combines the fluoride retention in oral cavity and the effects of CHX, i.e. reduction of plaque, gingival inflammation and *S. mutans* counts.^33^ In the present study, CHX0.05C containing sodium fluoride was in part more active than the CHX0.05 alone, which may support its use in preventing caries.

CHX0.09C was supplemented with hyaluronic acid. In dentistry, an adjunctive topical application may lead to additional clinical benefits in periodontal therapy.^6^ Hyaluronic acid, a glycosaminoglycan, is well known for its anti-inflammatory and wound-healing efficacy.^19^ Hyaluronic acid inhibits bacterial adhesion and biofilm formation.^27^ In the present study, CHX0.09C inhibited biofilm formation to a greater extent than did CHX0.12. Here, further research might be of interest to verify the role of hyaluronic acid as a component in mouthrinses.

In the present in vitro experiments, also a gel formulation containing 0.5% CHX was included, but the effect on bacterial counts was not superior to the use of 0.2% CHX solution. This is an agreement with findings of a systemic review that favoured mouthrinses to gels for clinical applications.^31^

The activity of CHX formulations and solutions was low on an already formed ‘periodontal’ biofilm. Only the highest concentrations of 0.2% CHX exerted some effect. This in vitro result may re-emphasise the general guidelines that mechanical removal of a biofilm by scaling and root planing is essential in initial therapy of periodontitis.^30^

## Conclusion

Taken together, the present in vitro data support an anti-biofilm activity of the novel CHX, Citrox, poly-L-lysine and xylitol oral-healthcare formulations. However, the biofilm inhibiting effect might not be related to Citrox, which cannot replace CHX in such products. Further studies are warranted to confirm the present findings in various clinical settings.
